# Case Report: Pseudotumor due to Trunnionosis in Ceramic‐on‐Ceramic THA Monoblock Acetabular Cup Disengagement

**DOI:** 10.1155/cro/6657448

**Published:** 2026-06-11

**Authors:** M. Olivieri, R. Guarracino, D. Lup, A. Maso, E. Storni, S. Squarzoni, V. Peccerillo, P. Cimatti, D. Dallari, P. Caldora

**Affiliations:** ^1^ Department of Orthopedics, Centro Chirurgico Toscano, Arezzo, Italy; ^2^ Laboratory of Microbiology and GMP Quality Control, IRCCS Istituto Ortopedico Rizzoli, Bologna, Italy, ior.it; ^3^ CNR Institute of Molecular Genetics “Luigi Luca Cavalli-Sforza”-Unit of Bologna, Bologna, Italy; ^4^ IRCCS Istituto Ortopedico Rizzoli, Bologna, Italy, ior.it; ^5^ Nursing, Technical and Rehabilitation Assistance Service, IRCCS Istituto Ortopedico Rizzoli, Bologna, Italy, ior.it; ^6^ Reconstructive Orthopaedic Surgery Innovative Techniques-Musculoskeletal Tissue Bank, IRCCS Istituto Ortopedico Rizzoli, Bologna, Italy, ior.it

**Keywords:** ceramic-on-ceramic THA, disassembly, early diagnosis, monoblock acetabular component, pseudotumor

## Abstract

Adverse local tissue reaction (ALTR) and pseudotumor formation, representing inflammatory responses to wear debris that lead to osteolytic lesions, remain major long‐term complications of total hip replacement. Hard‐bearing total hip prostheses with improved tribological properties—particularly large‐diameter head (LDH) ceramic‐on‐ceramic (CoC) bearings and preassembled cup designs—have been introduced into clinical practice to minimize wear, increase the range of motion (ROM), and reduce dislocation rates. We describe the case of a young asymptomatic patient with a CoC total hip arthroplasty (THA) featuring a monoblock acetabular component, in whom a pseudotumor was incidentally detected. The lesion was associated with trunnionosis following spontaneous liner disassembly, which occurred without any clinical symptoms or audible signs such as the typical hip “clunk.” This case highlights the diagnostic challenges in identifying periprosthetic adverse reactions in asymptomatic patients and emphasizes the importance of timely recognition to prevent technically demanding revision procedures in catastrophic implant failures.

## 1. Introduction

Adverse local tissue reaction (ALTR) is an uncommon but clinically significant complication following total hip arthroplasty (THA). It encompasses a spectrum of noninfectious, nonneoplastic inflammatory lesions, often presenting as granulomatous or cystic masses (pseudotumors) in the periprosthetic tissues. These lesions may initially manifest with pain or discomfort, but can also remain clinically silent. Progressive ALTR may lead to soft tissue destruction, osteolysis, and compromise of periarticular structures, ultimately resulting in implant failure and instability [1, 2].

ALTR and pseudotumor formation were initially described in association with metal‐on‐metal (MoM) bearings, where tribocorrosion at the articulating surfaces represents the principal source of metal debris. However, increasing evidence has demonstrated similar reactions in metal‐on‐polyethylene (MoP) and ceramic‐on‐polyethylene (CoP) constructs, implicating mechanically assisted crevice corrosion (MACC) at modular junctions—particularly the head–neck interface—as a key pathogenic mechanism independent of the bearing surface [3–5].

The introduction of ceramic materials has substantially improved the tribological performance of THA, reducing wear‐related debris and the risk of osteolysis. Advances in ceramic technology, including the development of alumina matrix composites, have enhanced mechanical strength and reliability, markedly lowering the incidence of component fracture. To further optimize joint stability and range of motion, large‐diameter head (LDH; > 36 mm) ceramic‐on‐ceramic (CoC) bearings have been adopted, while also mitigating wear and corrosion issues associated with MoM systems. In parallel, monoblock acetabular components with preassembled ceramic liners housed within a metal shell have been developed to reduce the risk of liner malpositioning and fracture.

Despite these advances, failure mechanisms in CoC THA are not fully understood, particularly in the context of nonarticulating interfaces [6–30].

We report the case of an incidentally detected pseudotumor in a young, asymptomatic patient with a CoC THA utilizing a monoblock acetabular component.

## 2. Case Presentation

**Figure 1:**
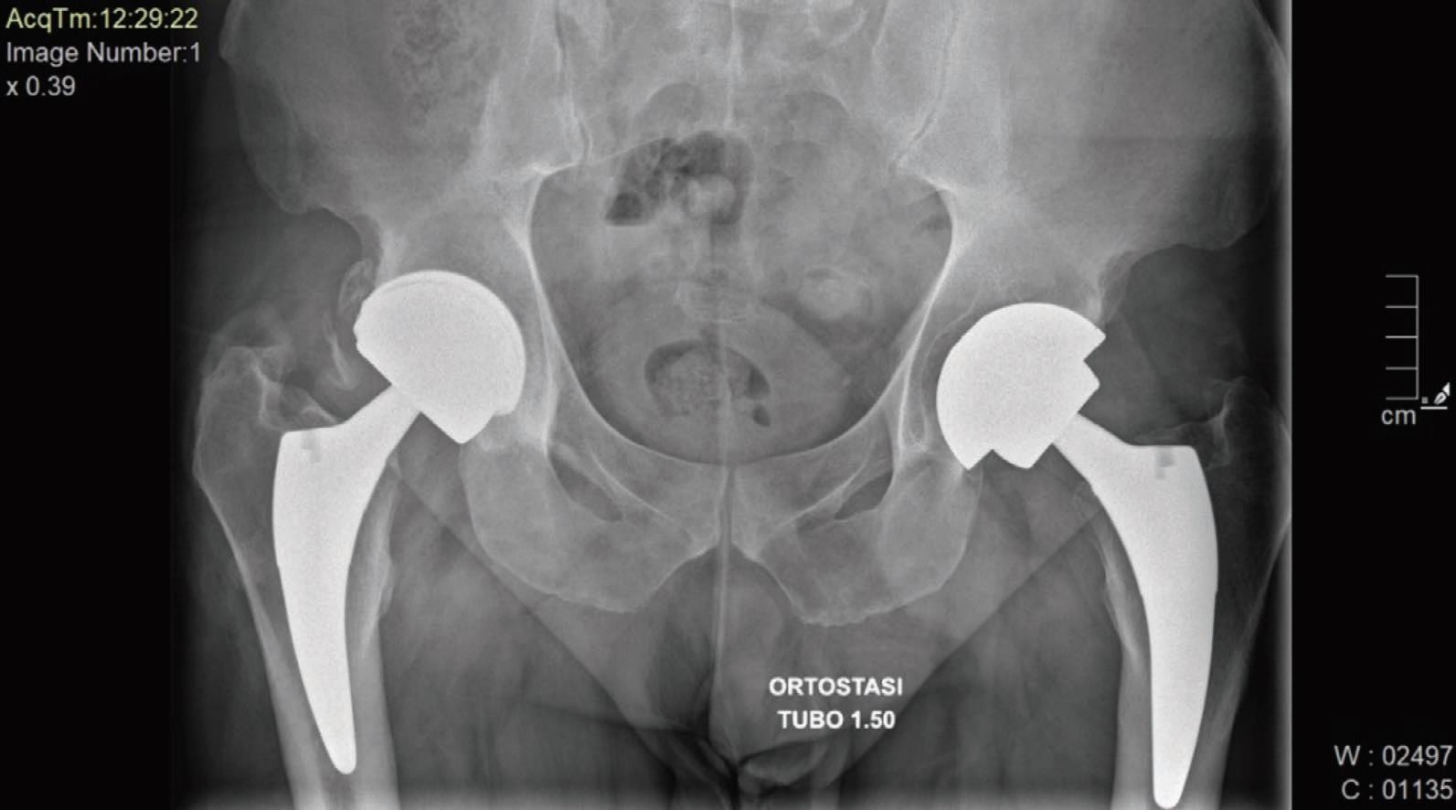
X‐ray: left THA postoperative control.

In 2015, a 43‐year‐old man (body mass index, 22.6 kg/m^2^) underwent right THA for idiopathic osteonecrosis of the femoral head. Given the patient′s young age and functional demands, a cementless CoC articulation was selected. A preassembled monoblock acetabular component (Maxera Hip System, Zimmer Biomet, Warsaw, Indiana, United States) was implanted, with a cup size of 56 mm and a 44‐mm femoral head. The femoral component consisted of a titanium (Ti) stem (Fitmore, size B6; Zimmer Biomet) with a proximally applied hydroxyapatite coating (Figure 1).

The postoperative course was uneventful, and the patient completed a standard rehabilitation protocol without complications, returning to full activity.

In 2021, 60 years after the index procedure, the patient presented with pain in the contralateral (left) hip. Magnetic resonance imaging (MRI), performed to evaluate suspected osteonecrosis of the left femoral head, incidentally revealed a heterogeneous fluid collection posterior to the right hip prosthesis.

At that time, the patient was completely asymptomatic with respect to the operated (right) hip. He denied any history of trauma, falls, or mechanical symptoms, including clicking, grinding, or audible “clunk.” There were no systemic symptoms such as fever, fatigue, or malaise.

Physical examination of the right hip demonstrated no swelling, erythema, or tenderness to palpation. Range of motion was full and pain‐free, without audible noise or squeaking during functional maneuvers, including sit‐to‐stand transitions. Abductor strength was normal and symmetric, with no evidence of Trendelenburg gait.

Radiographic evaluation showed a well‐positioned acetabular component, with no radiolucent lines, osteolysis, or interval changes compared with immediate postoperative imaging.

Quantitative assessment of hip motion demonstrated full extension, 95° of flexion, 35° of external rotation, and 10° of internal rotation in the seated position.

An infectious workup was unremarkable. Synovial fluid analysis revealed a leukocyte count of 204 cells/*μ*L, with 33.8% polymorphonuclear cells and 66.2% mononuclear cells, and a red blood cell count of 4000 cells/*μ*L. Serum inflammatory markers were within normal limits, with an erythrocyte sedimentation rate of 20 mm/h and a C‐reactive protein level of < 5 mg/L.

Given the progression of symptomatic osteonecrosis in the contralateral hip and the absence of clinical or laboratory signs suggestive of infection or inflammation in the right hip, the patient subsequently underwent left THA. A CoP articulation (Continuum system, Zimmer Biomet) with a Ti femoral stem (Fitmore, size B) was implanted.

The postoperative course was unremarkable. However, 6 months later, the patient began to experience discomfort and palpable swelling in the right gluteal region.

**Figure 2:**
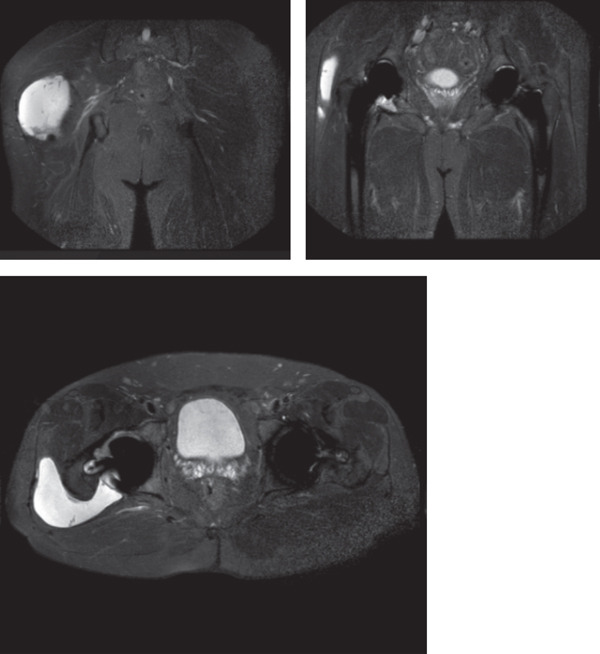
MRI of the right hip showing periprosthetic posterior fluid collection.

MRI revealed a nonhomogeneous fluid collection posterior to the right greater trochanter, located between the gluteus medius and gluteus maximus muscles, measuring approximately 50 mm in diameter (Figure 2).

**Figure 3:**
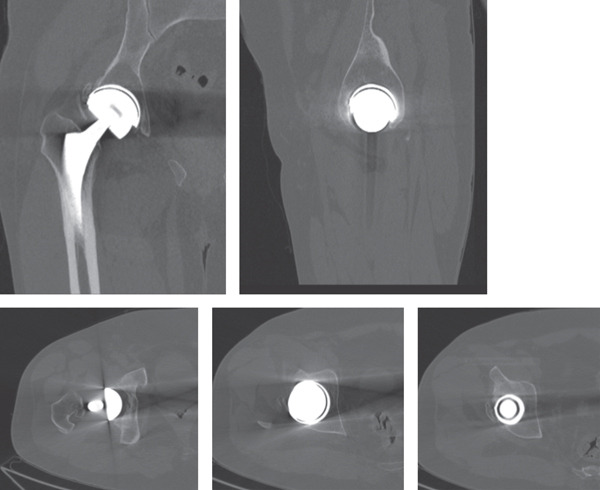
CT scan showing no signs of osteolysis and proper acetabular cup positioning.

A subsequent CT scan showed a well‐positioned acetabular component with correct inclination and anteversion, without evidence of osteolysis (Figure 3).

Aspiration of the collection yielded cloudy brown fluid with a white blood cell count of 2150 cells/*μ*L. Cultures were negative. The sample was referred to the Electron Microscopy Services of the IRCCS Istituto Ortopedico Rizzoli (Bologna, Italy) for synovial fluid microanalysis.

Approximately 200 *μ*L of synovial fluid was placed directly onto the glossy side of a polycarbonate filter (Isopore membrane filter, Millipore, Ireland) with a pore size of 0.2 *μ*m, which had been inserted into a filter holder. Synovial proteins were digested by at least three sequential additions of sodium hypochlorite and filtered through a 0.22 *μ*m membrane. Subsequently, 50 mL of double‐distilled sterile water was flushed through the filter, which was then air‐dried.

A central portion of the filter (approximately 8 × 8 mm) was mounted onto a scanning electron microscopy (SEM) stub using double‐sided adhesive tape, edged with silver conductive paint, gold‐sputtered, and examined with a Zeiss EVO MA10 electron microscope operated at 20 kV. Micrographs were taken at 10,000× magnification.

Quantitative evaluation was performed on 20 randomly selected fields, each measuring 90 *μ*m^2^ and representing all regions of the filter. The particles were counted, their major diameters measured, and their chemical composition determined by energy‐dispersive x‐ray (EDX) microanalysis. Analyses were conducted at 8.5 mm working distance using an Oxford INCA Energy 200 detector system [31–37].

Qualitative analysis of wear debris revealed Ti and aluminum (Al) fragments ranging in size from 2.5 to 34 *μ*m, displaying flake‐like morphology with sharp edges. A few calcium granules were also observed, appearing both as individual particles (approximately 2 *μ*m in diameter) and as aggregates up to 6 *μ*m. No Al fragments associated with ceramic material were detected.

These findings indicate the presence of prosthetic wear debris of metallic origin, characterized by variable particle dimensions and morphology, consistent with wear of the metallic components within the prosthesis.

After discussion of the risks and benefits and following informed consent for research purposes, the patient elected to undergo revision THA. During revision surgery—performed through the prior surgical approach—no purulence was identified within the joint; however, dark, turbid synovial fluid fountained upon arthrotomy. Samples were sent for differential cell count, culture, and histologic analysis of the hip capsule.

**Figure 4:**
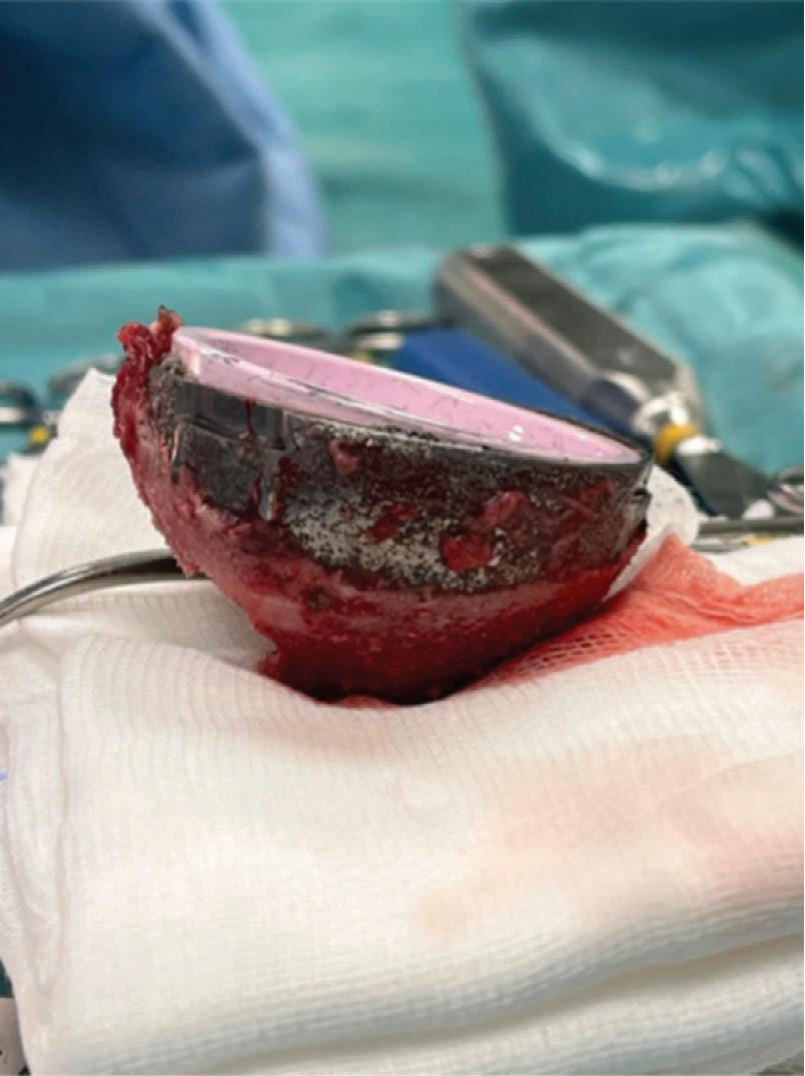
Intraoperative photograph clearly showing the disassembly of the ceramic liner with respect to the metal back.

Intraoperative evaluation of hip stability demonstrated femoral neck–liner contact during extension beyond neutral and external rotation. Upon dislocation of the prosthesis, the capsule and synovium appeared abnormal, and a fluid collection was noted as the abductors were elevated from the greater trochanter. Findings were consistent with a typical ALTR, characterized by synovial necrosis and metallosis along the edge of the monoblock component, without evidence of increased inflammatory cell infiltration, abscess formation, or vasculitis. Inspection revealed partial disassembly of the ceramic liner relative to the metal backing, with a tilt of several degrees. The liner was misaligned but not mobile (Figure 4).

The findings confirmed liner dissociation and trunnion wear due to impingement, without evidence of ceramic or metal fracture. Multiple soft tissue and bone samples were collected for microbiological and histological evaluation. No bacteria were isolated, and histology showed no acute inflammation.

**Figure 5:**
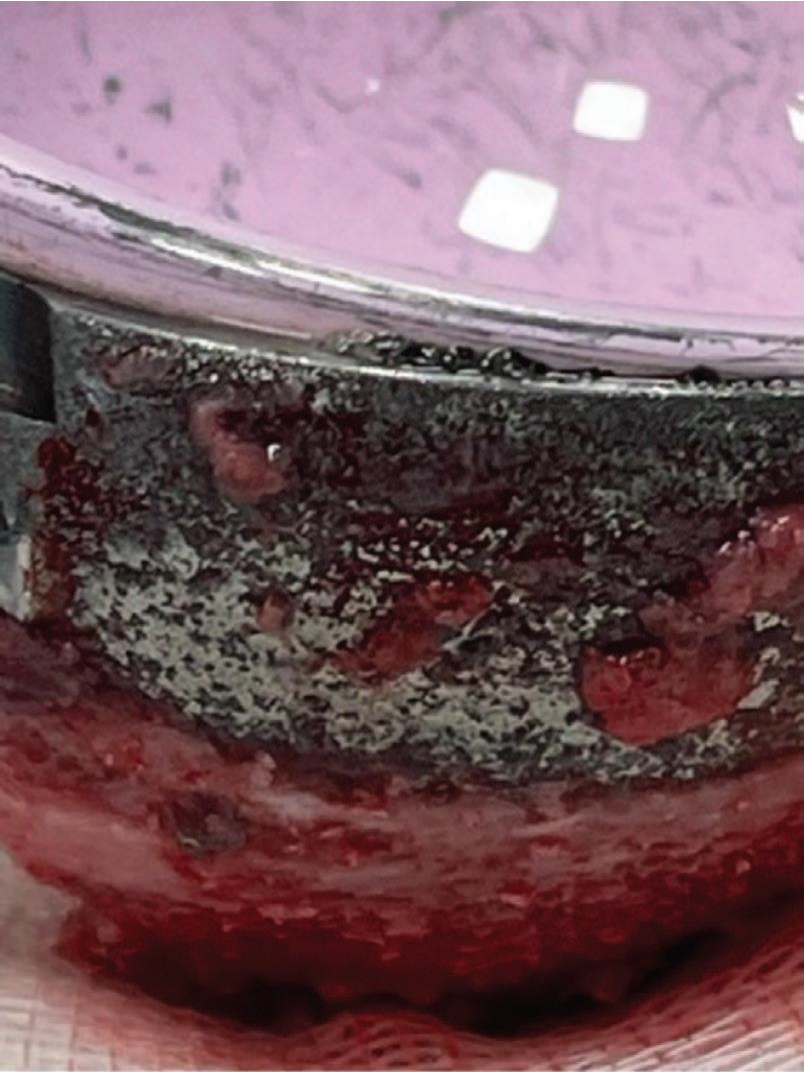
Metal residues from fracking corrosion, predominantly titanium.

**Figure 6:**
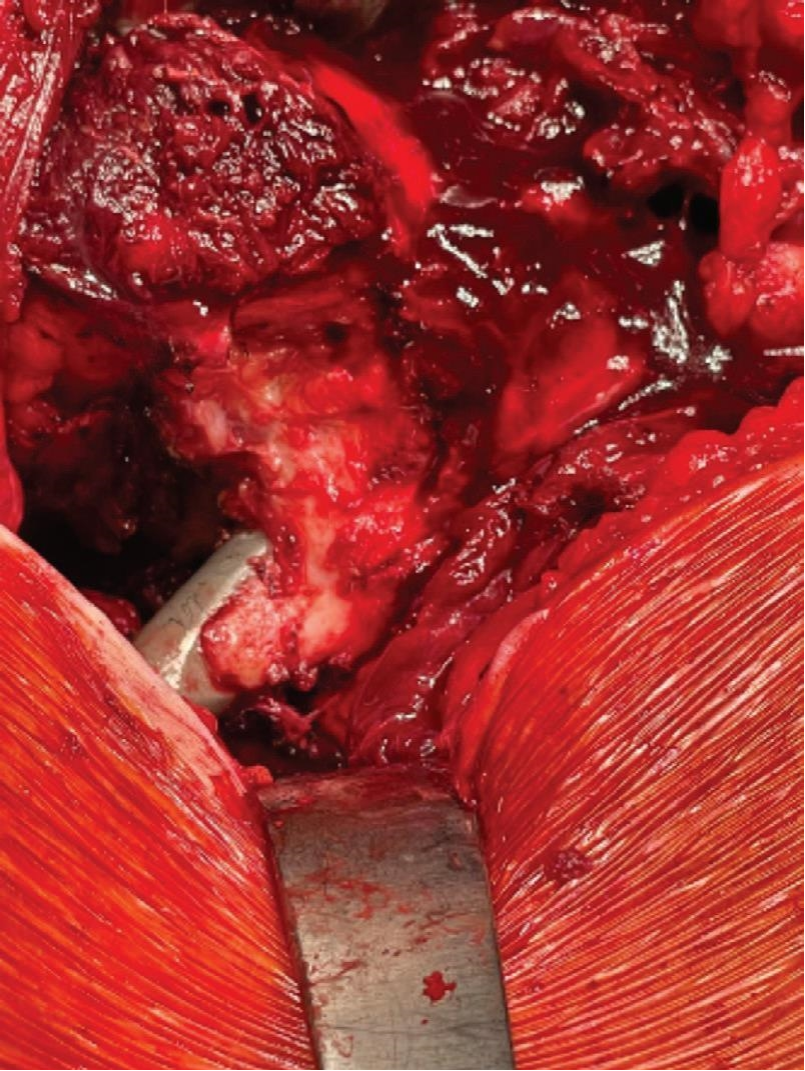
Appearance of the proximal femur with necrosis and pseudotumor area.

Inspection of the femoral trunnion revealed minimal damage despite component‐to‐component impingement, allowing retention of the femoral stem and avoidance of unnecessary morbidity associated with its removal. Metallic debris consistent with trunnionosis, predominantly Ti, was identified around the interface (Figure 5). The proximal femur exhibited localized necrosis and pseudotumor formation, confirming the presence of an ALTR (Figure 6).

The well‐fixed acetabular component was removed with minimal bone loss. After reaming and curettage of necrotic tissue, the cavity was thoroughly irrigated.

**Figure 7:**
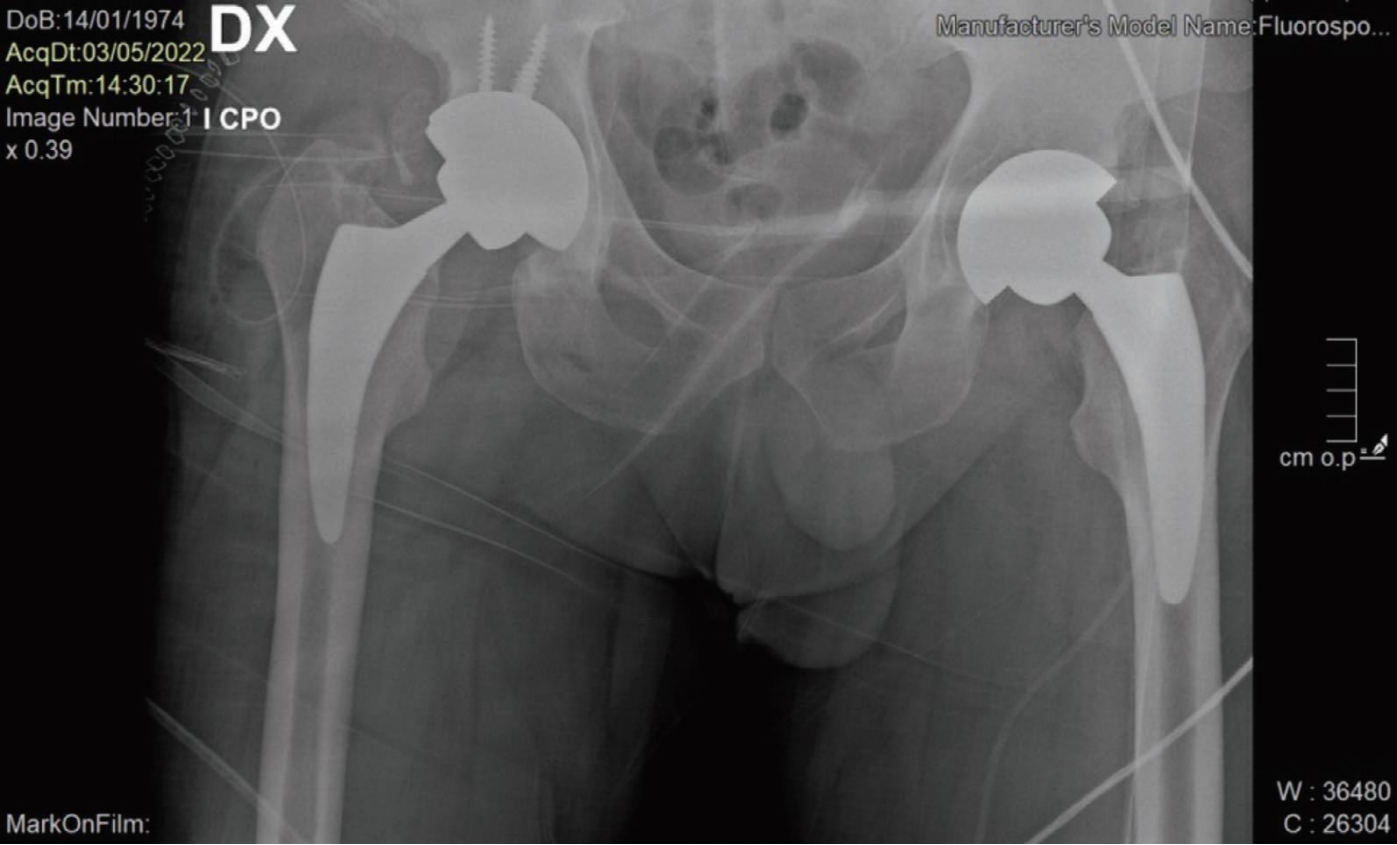
Postoperative x‐ray of right THA revision.

Revision THA was performed using a 62‐mm TMT acetabular shell (Zimmer Biomet, Warsaw, Indiana, United States) secured with three posterosuperior bone screws. A 36‐mm polyethylene liner (+10°) was implanted, combined with a +0 mm BIOLOX Option femoral head (CeramTec, Plochingen, Germany) placed on the existing femoral trunnion. Intraoperatively, the construct demonstrated excellent stability and a full range of motion without impingement (Figure 7).

The patient′s postoperative course was uneventful. Rehabilitation began on postoperative Day 1, and he was discharged home on Day 4. At the 8‐month follow‐up, the patient remained asymptomatic, with full function and no complications.

## 3. Discussion

The present report describes, to our knowledge, a rare documented case of confirmed trunnionosis secondary to spontaneous liner dissociation in a CoC THA with a monoblock acetabular component, occurring in an asymptomatic patient without audible hip noise. This presentation differs from previously reported CoC cases, which predominantly describe pseudotumor formation or ALTRs in the absence of taper damage [8, 27, 28].

Existing literature on CoC THA identifies three principal patterns of complications: (i) soft tissue reactions without evidence of corrosion, (ii) ceramic fracture or third‐body wear, and (iii) atypical cases attributed to alternative mechanisms such as impingement or infection. Notably, prior reports have consistently documented an intact trunnion without macroscopic or electrochemical evidence of corrosion, thereby excluding true trunnionosis. Accordingly, trunnionosis remains primarily associated with MoP and MoM bearings, where MACC at the head–neck junction is well established [9–11, 25, 27, 28, 34–38].

Trunnionosis is characterized by fretting and corrosion at the modular head–neck interface, leading to metallic debris release, local inflammation, and potential structural compromise. The underlying mechanism involves cyclic micromotion at the taper junction, which disrupts the protective oxide layer and promotes electrochemical degradation. This process results in the generation of metal ions and particulate debris, which induce cytotoxic and proinflammatory responses, including aseptic lymphocyte‐dominated vasculitis‐associated lesions (ALVALs) and, in some cases, pseudotumor formation. The use of LDHs further amplifies these effects by increasing torsional and bending stresses at the trunnion.

In contrast, CoC bearings are characterized by excellent tribological performance, with minimal wear and low friction due to the intrinsic material properties of modern alumina matrix composites. Consequently, the primary articulating surfaces are unlikely to represent a significant source of debris under normal conditions. However, failure at nonarticulating interfaces may introduce alternative pathogenic pathways.

In the present case, the monoblock acetabular component incorporated a preassembled ceramic liner within a Ti shell. We hypothesize that early, subclinical liner dissociation led to micromotion at the liner–metal back interface. This instability may have promoted local fretting and corrosion at the Ti interface, generating metallic debris despite the CoC articulation. Progressive liner displacement likely resulted in edge loading and intermittent impingement between the femoral neck and the partially disengaged ceramic liner. This abnormal contact could have further exacerbated mechanical wear and contributed to taper damage at the head–neck junction, ultimately leading to trunnionosis.

This proposed mechanism highlights a dual‐source process: (i) corrosion at the liner–shell interface secondary to micromotion and (ii) mechanically induced damage at the trunnion due to altered joint kinematics and impingement. The absence of noise or symptoms in our patient suggests that liner dissociation may occur insidiously, without the characteristic “clunk” described in prior reports, thereby delaying diagnosis.

Although rare, inflammatory reactions in CoC THA have been described even in the absence of metallic components, suggesting that both ceramic and metal‐derived debris may contribute to ALTR. Malpositioning, particularly excessive cup abduction, may further predispose to edge loading and abnormal stress distribution, compounding the risk of mechanical failure.

From a clinical perspective, the diagnosis of pseudotumor remains challenging, particularly in asymptomatic patients. Imaging typically demonstrates well‐defined fluid collections with a low‐signal‐intensity rim on MRI, although differentiation from infection, loosening, or other periprosthetic complications is essential. The absence of elevated metal ion levels, pain, or audible phenomena may further obscure early recognition, as observed in this case.

Management of pseudotumor associated with trunnionosis is complex and associated with relatively high complication rates following revision surgery. Thorough debridement of affected tissues is critical. In selected cases, retention of a well‐fixed femoral stem with the use of a Ti sleeve may be considered if taper damage is limited, thereby reducing surgical morbidity. Replacement of cobalt‐containing components with ceramic or Ti alternatives is recommended to minimize further metal ion release.

In summary, this case expands the current understanding of failure mechanisms in CoC THA by demonstrating that trunnionosis may arise indirectly from liner dissociation in monoblock systems. It underscores the importance of considering nonarticulating interfaces as potential sources of debris generation and highlights the need for heightened vigilance even in asymptomatic patients.

## 4. Conclusion

To our knowledge, this report describes a rare documented case of confirmed trunnionosis secondary to spontaneous liner dissociation in a CoC THA with a monoblock acetabular component. Notably, this occurred in an asymptomatic patient without audible hip noise and was identified incidentally in association with an ALTR/pseudotumor.

This case demonstrates that, even in contemporary LDH CoC designs with preassembled liners, mechanical complications and interface corrosion may develop insidiously, in the absence of pain or characteristic mechanical symptoms. Clinicians should therefore maintain a high index of suspicion for atypical presentations, particularly in patients at risk for component micromotion or trunnion wear.

Early detection through appropriate imaging and intraoperative assessment is essential to limit progressive soft tissue damage. Surgical management may require revision of modular components and extensive debridement; however, retention of a well‐fixed femoral stem, combined with the use of a protective sleeve, may reduce surgical morbidity in selected cases.

This report underscores the importance of continued surveillance and systematic reporting of rare complications in CoC THA, with implications for both implant design optimization and clinical decision‐making.

## Funding

No funding was received for this manuscript.

## Disclosure

All authors reviewed and approved the manuscript.

## Ethics Statement

The patient provided written informed consent for the publication of this case report and any accompanying images. All data were anonymized to ensure the protection of patient privacy. All procedures were conducted in accordance with institutional ethical standards and applicable regulations.

## Conflicts of Interest

The authors declare no conflicts of interest.

## Data Availability

The data that support the findings of this study are available from the corresponding author upon reasonable request.
